# Udder health, conceptual construct, and uses of the term: A systematic review from 1962 to 2019

**DOI:** 10.14202/vetworld.2022.855-869

**Published:** 2022-04-08

**Authors:** Richard Zapata-Salas, José F. Guarín, Leonardo A. Ríos-Osorio

**Affiliations:** 1 School of Microbiology, University of Antioquia, Medellín, Antioquia, Colombia; 2 Research Group in Health and Sustainability, Research Group in Veterinary Microbiology, University of Antioquia, Medellín, Antioquia, Colombia; 3 Department of Agricultural Sciences, University of Antioquia, Medellín, Antioquia, Colombia; 4 Research Group in Agricultural Sciences – GRICA (Acronym in Spanish), University of Antioquia, Medellín, Antioquia, Colombia

**Keywords:** conceptual categories, culture, livestock productivity, politics, public health, udder health

## Abstract

**Background and Aim:**

Udder health management is essential for the further development of milk production systems and public health. This process depends on the generation of knowledge regarding control, prevention, and promotion of health. In scientific literature, it is impossible to find a synthesis of the categories that would allow comprehension of the complex phenomenon udder health. Different research approaches have allowed this polysemic concept, described by some researchers as multifactorial and by philosophical perspectives as a social phenomenon, to be further studied. Thus, the objective of this systematic review was to systematize the conceptual categories of udder health and the use of the term in the original articles published in the scientific literature from the period 1962 to 2019.

**Materials and Methods:**

A systematic review with a broad approach was designed by applying the phases of identification, screening, selection, and inclusion criteria described in the Preferred Reporting Items for Systematic Reviews and Meta-Analyzes guide. An exhaustive search of original articles by specificity was carried out in the Science Direct, PubMed, Scielo, LILACS, and Google Scholar databases. The investigation was carried out on November 22, 2019. According to the inclusion criteria established, articles needed to be original studies, to be publications on bovine livestock, written in English, Spanish and Portuguese. Furthermore, the articles considered needed to tackle the term udder health so that its conceptual categorization could be extracted. Google Scholar patents and citations and articles removed from databases or not available were excluded from the study and those that, based on the reading of the complete text, considered the farming of animal species other than bovine. A qualitative synthesis of the year of publication, continent, approach, type of study, and conceptual category of udder health was carried out by calculating frequencies (Statistical Package for the Social Sciences version 24).

**Results:**

In total, 165 articles were included in the study. Eight conceptual categories, consolidated over time, were systematized, showing that udder health is not a static problem, and that science has been responding through the generation of new knowledge around conceptual categories as different udder health problems emerge.

**Conclusion:**

Culture and politics were two categories, related to all the others, that stood out in the results. These two categories were of great interest in countries advanced in milk production and in the implementation of udder health policies, which acknowledge the producer and other actors of the production chain as fundamental political actors for policies, decision-making processes, and public health care to be effective. The lack of synonyms for the term udder health (e.g., mastitis) may have led to the exclusion of important articles in each category. However, the constriction to the term udder health was intentional and aimed at constructing the concept. Udder health is hereby understood as a health-disease process, different from the term mastitis, which from its semantic origin, refers only to the disease process. According to this study, the concept can be understood through the categories of traditional epidemiology based on risk factors and disease; microbiology; genetics, resistance, and immunity; animal welfare; nutrition; organic production; culture; and politics.

## Introduction

Udder health is considered a polysemic concept given the use of the term and the various conceptual categories found in scientific journals from different parts of the world. Over time and for several decades, udder health was studied from the perspective of traditional epidemiology, based only on the analysis of its risk factors. However, since the 1960s, additional conceptual categories have been incorporated, allowing the conceptual restructuring of the concept, changing its definition, and acknowledging it as a complex phenomenon [1-8]. The conceptual construction of this health-disease phenomenon has allowed plans, programs, and campaigns for mastitis control and udder health promotion to be designed and implemented. The introduction of 5-point plan in the early 1960s, which proposed actions to control clinical and subclinical mastitis based on simple strategies focused on technical processes related to milking and disease recording, is an example of this. Years later, 10-point plan incorporated other fundamental elements for prevention, such as environmental pathogens control, the establishment of goals aimed at udder health, its regular monitoring, and periodic review of mastitis control programs [9]. Despite the introduction of these complementary control strategies, the overall management of udder health has had to evolve and become even more complex. The complex nature of udder health problems requires the understanding and consideration of new and diverse conceptual categories. In recent years, successful studies and programs for udder health care (e.g., Australia’s Countdown Downunder, New Zealand’s Smart SAMM Plan, the Dutch Udder Health Program, the Norwegian Program of Mastitis Control, and the DairyCo Mastitis Control Plan of Great Britain) have acknowledged that for the implementation of a national udder program to succeed, the need for communication between actors in the dairy chain, active participation of producers, and teamwork with other actors are essential. These challenges have been thus consolidated as conceptual categories of udder health management, focused on actors, and cultural-political processes related to the dairy industry [7,10-12].

Consequently, it is essential to systematize the uses of the term udder health and the conceptual categories described in the scientific literature to organize the theoretical elements that define the concept according to the current reality of the phenomenon. The impact of udder health problems on animal health and welfare, livestock productivity, public health [13,14], and the high availability of studies support the importance of conducting a systematic review using Cochrane’s comprehensive approach. This approach establishes a practical method to synthesize the scientific production related to a specific area of interest, update a field of knowledge, summarize the available evidence, and analyze the possibilities of generalization of the published information, among other uses valuable for researchers in the area of knowledge and the affected community [15,16].

Therefore, the study aimed to systematize the conceptual categories of udder health and the use of the term, as it appears in the original articles published in the scientific literature during the period 1962-2019.

## Materials and Methods

### Ethical approval

This study was approved by the Bioethics Committee of the University Research Headquarters at the Universidad de Antioquia (Sede de Investigación Universitaria - SIU), Approval Act, 19-101-876, according to: Resolution 8430 de 1993 of the Colombian Ministry of Health; the principles expressed in the Declaration of Helsinki; the Health and Human Services regulations for the protection of human subjects in research at the Code of Federal Regulations, Title 45, Part 46; the National Health Institutes of the U.S. Department of Health and Human Services (1991); and Resolution 2378 of 2008, issued by the Colombian Social Protection Ministry.

### Study period

The search was conducted on November 22, 2019, and no time restrictions were applied retrospectively.

### Type of study

The type of study was a systematic review. This study was conducted following the Preferred Reporting Items for Systematic Reviews and Meta-Analyzes and applying the identification, screening, selection, and inclusion phases described in the guide [17].

### Identification

An exhaustive search of original articles was carried out by specificity in the Science Direct, PubMed, Scielo, LILACS, and Google Scholar databases, using four search strategies according to the combination of the terms “udder health” and “bovine,” “cow,” “dairy,” and “cattle.” Since the term udder health has been emerging lately as a concept in dairy science, but its conceptualization has not yet been completed, the papers considered for this systematic review were selected using a broad approach that allowed the characterization of the conceptual use of the term udder health in scientific research without incorporating other synonyms. Even when the lack of synonyms for the term (e.g., mastitis) may lead to the exclusion of important articles in each category, focusing exclusively on the term udder health was an intentional choice aimed at constructing the corresponding concept, which we understand as a health-disease process. The term mastitis was not considered since it refers semantically only to the disease process. The search was carried out on November 22, 2019. Some syntaxes used were: TITLE (udder health) and TITLE-ABSTR-KEY (bovine), (“udder health” [Title]) AND (dairy [Title/Abstract]), (ti:(“udder health”)) AND (ab: (bovine)).

### Screening

Articles containing the search terms were considered eligible when, in Science Direct, the term udder health was part of their title and the other words were present in title, abstract, or keywords. In PubMed, articles considered contained the term udder health in the title and the other words in the title or synopsis. In Scielo, articles considered contained the term udder health in the title and the other words in the abstract. In LILACS, articles considered were those with the term udder health in the title and the other words in title, abstract, or subject. In Google Scholar, articles considered were those with all terms in the title. According to the inclusion criteria, to be eligible, an article needed to be an original study, to be a publication on bovine livestock, written in English, Spanish, or Portuguese, and to be related to the study of udder health so that the use of the term udder health and its conceptual categorization could be extracted.

### Selection

Quotations and Google Scholar patents were excluded from the search. Articles that were removed from the databases or that were not available were excluded from the study. Based on the reading of the complete text, articles that studied species different from cattle were excluded from the study. In addition, bibliographic references of the included studies were reviewed to expand search results and include additional relevant references.

### Inclusion

After completing the phases above, a qualitative synthesis of the resulting articles was conducted based on the extraction of the variables title, authors, year of publication, continent, approach, type of study, and conceptual category of udder health. Three authors applied the search protocol, selected the articles, and extracted the variables to guarantee reproducibility. It was previously agreed that discrepancies would be resolved by consensus. The compiled articles were stored in an Excel database, and duplicates were eliminated. Statistical Package for the Social Sciences software version 24 (IBM Corp., NY, USA) was used for the qualitative synthesis through frequency calculation.

## Results and Discussion

**Figure-1:**
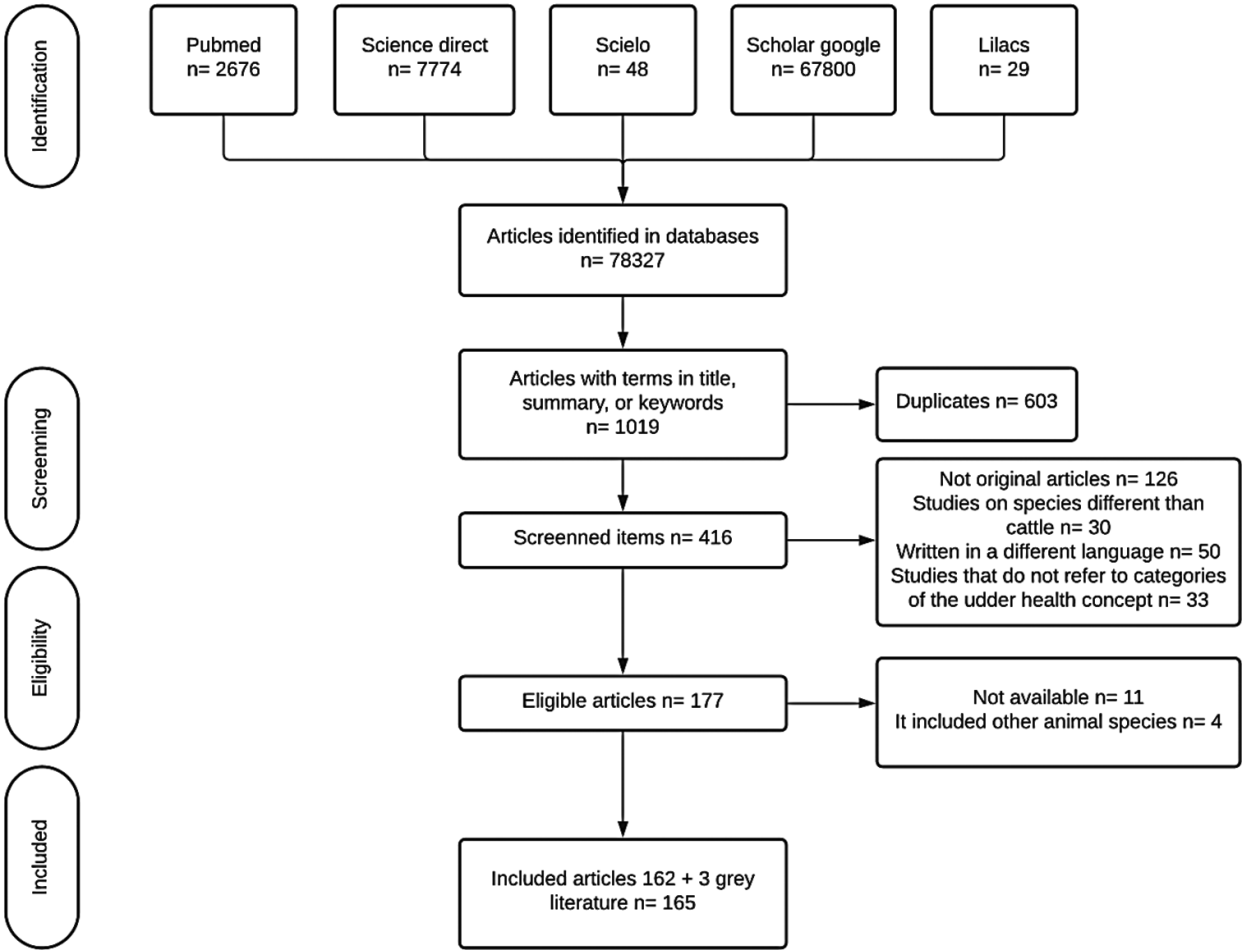
Manuscript selection flowchart.

A total of 78,327 articles were obtained from the initial search using the routes described in the Methods section. When restricted by title, abstract, or keywords according to the options of each source, 1019 articles were selected to continue. After applying the inclusion criteria, 603 duplicates and 239 articles were excluded. Furthermore, 15 articles were excluded during the selection phase. Three articles from grey literature were included in the study based on relevance for the systematic review and compliance with the selection criteria. In total, 165 articles were included in this study (Figure-1).

Most of the 165 articles were published in Europe and America, with 64.9% and 23.6%, respectively (Table-1). In the 44 countries that registered publications on udder health, the highest number of papers came from the United States (12.7%), the Netherlands (8.5%), Sweden (7.9%), Germany (7.3%), Denmark (4.8%), and Switzerland (4.8%) (data not shown in the table). Most articles were published between 2010 and 2019 (60.7%), primarily in regions that have made significant advances in programs and policies regarding udder health (Table-1).

**Table 1:** Temporal, spatial characteristics and by type of study of udder health research.

	n	%
Publications by period		
Studies between 1962 and 1969	2	1.2
Studies between 1970 and 1979	2	1.2
Studies between 1980 and 1989	8	4.8
Studies between 1990 and 1999	8	4.8
Studies between 2000 and 2009	45	27.3
Studies between 2010 and 2019	100	60.7
Continent of origin of the study		
Africa	7	4.2
America	39	23.6
Asia	9	5.5
Europe	107	64.9
Oceania	3	1.8
Research focus of the study		
Qualitative	2	1.2
Quantitative	163	98.8
Type of study		
Cases and controls	1	0.6
Cohort	1	0.6
Quasi-experimental	1	0.6
Longitudinal descriptive	16	9.7
Retrospective descriptive	4	2.4
Cross-sectional	73	44.3
Clinical study	1	0.6
Experimental	66	40
Grounded theory	2	1.2

Most studies were quantitative (98.8%), among which the cross-sectional descriptive studies and experimental studies were the most frequently published (Table-1). It is worth noticing that only two articles were qualitative. These were based on grounded theory and aimed at understanding human processes related to udder health (Table-1).

Eight conceptual categories were used to classify the concept of udder health found in the included publications (Table-2). Most of these studies were classified under the category traditional epidemiology based on risk factors and disease, the category microbiology, and the category genetics, resistance, and immunity as components that contribute to prevention. The conceptual categories culture (cultural aspects) and politics emerged in our results, especially in articles from the past decades. These conceptual categories comprise dairy farmers and other actors of the dairy industry as subjects of study. At the same time, they comprise animal welfare, nutrition, and organic production as elements to understand health promotion (Table-2).

**Table 2:** Conceptual categories of the udder health concept.

Conceptual category	n	%
Animal welfare	2	1.2
Culture	6	3.6
Traditional epidemiology based on risk factors and disease	65	39.4
Genetic, resistance, and immunity	28	17
Microbiology	31	18.8
Nutrition	14	8.5
Politics	10	6.1
Organic production	9	5.5

**Figure-2:**
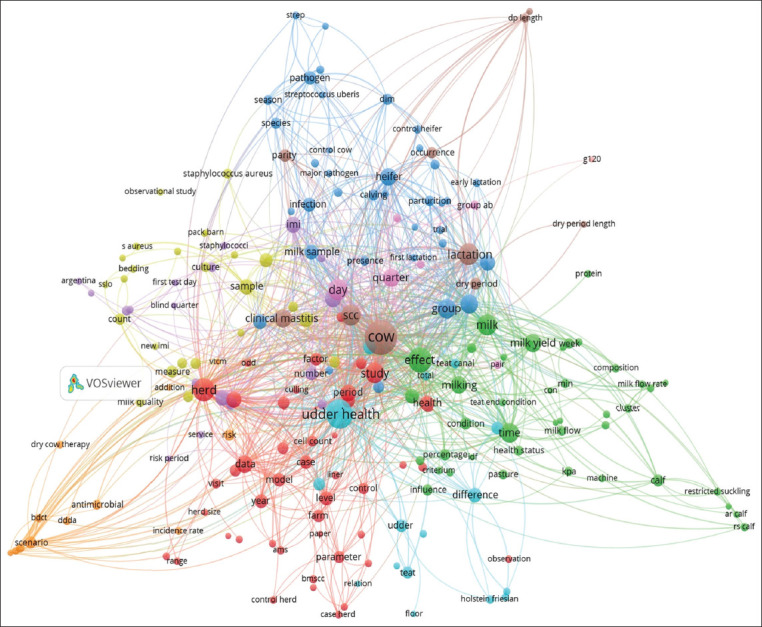
Network of keywords in publications focused on traditional epidemiology based on risk factors and disease and microbiology conceptual categories of the term udder health.

In the timeline studied in this paper, the first attempt to understand the concept of udder health was the conceptual categorization of the disease and its risk factors. This category comprised the theoretical elaboration of the disease process, that is, the study of alterations of udder health focused on the conditions that lead to bovine mastitis. In this conceptualization, udder health alterations were related to deficiencies in production systems practices, health practices, and the environmental and infrastructure facilities on the farm. Later in the timeline, a relationship between all of the mentioned risk factors with microbiology, another conceptual category of udder health, was identified. Figure-2 shows the keyword networks related to traditional epidemiology based on risk factors and disease, and microbiology obtained through the VOSviewer program. 1.6.16, 2020 (https://www.vosviewer.com).

In this systematic review, hygiene, as a management practice was found to be the object of study in the articles classified under the conceptual category Traditional epidemiology based on risk factors and disease. Some articles in this category highlighted the advantages that automated milking systems possess over manual milking systems [18], the access to pasture according to climatic conditions and environmental hygiene [19,20], and farm infrastructure as a protective factor against mastitis [21,22]. Furthermore, within this category, it was described that those cows with more than 90 days in milk (DIM), cows with two or more calvings, cows that do not get dry cow therapy, farms that do not perform post-milking teat sealing, and the age of the milkers (milkers over 50 years) are associated with greater bulk tank somatic cell count (BTCSS) [23]. Other studies found that prepartum feeding of concentrates can affect the udder health in primiparous cows [24] and that manure-free materials, frequent change, and bed cleaning favor udder health [25-27]. In addition, it has been identified that cows in confined housing systems had a higher bacterial count compared to cows from rotational grazing systems [28].

It has been found that defective milking machines and inappropriate milking management explain between 16 and 45% of the variation in udder health between herds [29]. It has also been proven that when equipment in good conditions is used during over-milking the rate of new infections in the udder does not increase [30]. Milking frequency has also been a matter of interest. Studies have compared milking frequencies of 6 and 3 times a day, and 3 and 2 times/day, finding that higher frequencies favor lower somatic cell count (SCC) [31,32]. However, these results are controversial since, in the long term, excessive milking could cause hyperkeratosis and make cows more prone to mastitis [33]. Other milking practices such as post-milking teat sealing, dry teat milking, milking machine disinfection, and pulsation system maintenance are associated with lesser BTSCC, that is, favor udder health [34]. Another controversial practice is restricted sucking, which instead of negatively altering health indicators of the udder [35] seems to improve them [36]. Characteristics of the anatomy and morphology of the udders and teats have also been the object of study in the udder health field. The sum of these studies has allowed the construction of theoretical elements to define checklists for cow selection, aimed at helping improve udder health in the herd [37-39]. The general health conditions of cows in production are related to udder health. For example, it has been described that lameness in cows increases the chances of mastitis appearance and a greater SCC [40], and likewise, self-sucking, a behavior associated with stress in the lactating cow, can cause failures in teat hygiene and udder structure [41].

The category microbiology includes the concept of udder health based on etiological agents, the use of antibiotics, and the susceptibility of the hosts. The susceptibility of cows to mastitis when they have coinfections has already been studied in relation to the bovine viral diarrhea virus [42,43]. It has been identified, in several studies, that in addition to the bacteria commonly found, *Staphylococcus aureus*, *Streptococcus agalactiae, Streptococcus dysgalactiae* [44], and Coagulase Negative Staphylococci (CNS) [45], are the main etiological bacterial agents of bovine mastitis. A study in Belgium reported 19 species of the *Staphylococcus* group, *Staphylococcus chromogens*, *Staphylococcus sciuri*, and *Staphylococcus* *cohnii*, as the predominant species that caused intramammary infection (IMI) in heifers and dairy cows [45]. A study in Belgium ensures that special attention should be paid to the CNS group, especially to *S. chromogens*, *Staphylococcus simulans*, and *Staphylococcus xylosus* due to their substantial effect on SCC. These are comparable to *S. aureus* effects, and their ability to generate persistent infections, requiring identification at the species level by a microbiology laboratory [46].

Similarly, in Dutch farms with greater SCC than the average for the study, *Streptococcus uberis* was found alongside CNS and *S. aureus* as the most frequent pathogens [21]. In South Africa, an 11-year retrospective study found CNS (60.96%) to be the most isolated bacteria from milk samples of lactating cows, followed by *S. aureus* (25.1%), *S. agalactiae* (5.92 %), *S. dysgalactiae* (2.27%), *S. uberis* (2.25%), *Enterococcus faecalis* (1.77%), and other bacteria (1.29%) [47]. The former, together with bacteria registered in other studies in lesser percentages [48], difficult culture bacteria, and groups with slower culture growth (e.g., *Mycoplasma bovis*) [49], were considered relevant in the decision-making process. Despite the importance of these emerging pathogens, recommendations to improve udder health are generally based on the most common pathogens found in the herd, rather than including specific herd circumstances and microbiological results [48].

The mammary gland is one of the anatomical sites that were believed to be sterile until recently. Consequently, it was early believed that a protective effect was caused by populations of beneficial bacteria competing against pathogenic bacteria that try to colonize the udder. The evolution of molecular methods has made it possible to understand that animals host a wide diversity of microbial communities. These microbial communities have evolved beside animals, creating complex, and mutualistic interactions. This relationship plays a decisive role in the biology and health status of the host. Some commonly found bacteria genera in healthy udders are *Ralstonia*, *Pseudomonas*, *Sphingomonas*, *Stenotrophomonas*, *Psychrobacter*, *Bradyrhizobium*, *Corynebacterium*, *Pelomonas*, *Faecalibacterium*, unclassified *Lachnospiraceae*, *Propionibacterium*, *Aeribacillus*, *Lactobacillus*, and *Paenibacillus*. The category microbiology, which comprises the microbiota of the udder, has recently become more relevant, mainly since the identification of these hosted bacterial groups could potentially define therapeutic alternatives against specific pathogens and rationalize the use of antibiotics in farms [50].

The ability of *S. aureus* to form biofilms in the mammary gland and milking equipment has been of great concern. A study described the sources of milk contamination by *S. aureus* in a commercial dairy farm in Chile. The results showed that biofilms of this bacteria on surfaces of milking equipment may play a role as a source of *S. aureus*, for both bulk tanks and udder contamination with the pathogen [51].

Dry cow therapies have been considered a preventive management practice against mastitis, although the results among studies can be variable. One investigation reported that a dry period between 30- and 60-days results in a lesser SCC in the subsequent lactation [52]. Similar results were obtained in another study in the Netherlands, where 600 mg cloxacillin was used during 8-10 weeks prepartum for cow drying, which resulted in a reduced number of positive samples in milk cultures at the time of parturition and between 10 and 14 DIM [53]. In Sweden, the incidence of mastitis during the first 12 weeks after calving tended to be greater among cows subjected to a 4-week dry period than an 8-week one [54]. Another research in Austria [55] found that peripartum antibiotic treatment of heifers with penethamate hydride was an effective way to protect the udder from IMI caused by *S. aureus* in the 1^st^ week postpartum and to minimize new IMI by *S. aureus* for 21 days postpartum [55]. A study carried out in the United States showed that the combination of intramammary sealant and subsequent intramammary antibiotic application during the dry period decreased the incidence of clinical and subclinical mastitis and the SCC throughout lactation, improving milk quality and udder health [56]. However, drying dairy cows with antimicrobials affects udder health, antimicrobial use, and economy, and non-quantifiable parameters [57]. Some of these non-quantifiable parameters, as concluded by the authors, are animal welfare and the practical achievability of goals on dairy farms. Therefore, depending on the importance of these parameters, optimal selection criteria for treatment should be carefully chosen to implement a selective dry cow therapy program [57].

The transition from a blanked dry cow therapy to a selective dry cow therapy in the Netherlands reduced the number of antimicrobials used in dairy herds without deleterious effects on udder health during the dry period [58]. This study adds to the body of evidence showing that in general, when cows and herds are technically managed, animals at low risk of developing IMI during the dry period can be successfully dried without the use of intramammary antimicrobials [58]. Supporting this strategy, which, in turn, decreases the risk of bacterial resistance, a study in the United States demonstrated that the use of a culture-guided selective dry cow therapy program resulted in postpartum udder health comparable to a blanked dry cow therapy program while reducing the use of antibiotics in the dry period by 48% [59].

Articles with successful results in drying antibiotics are commonly published; however, their results are not always consistent. A study in the United States found that intramammary antibiotic treatment at calving did not reduce the number or any specific bacterial type of intramammary infections 30 days postpartum [60]. This indicates that antibiotic treatment did not cure or prevent intramammary infections in early lactation [60]. Adequate mastitis management can help reduce the consumption of antimicrobials without negative implications for milk quality and udder health. This could be beneficial due to the importance these antibiotics have for human health [61].

Several studies state that udder health is a complex phenomenon that could vary depending on multiple conditions, that correction of a risk factor or elimination of bacteria in a mastitis case does not necessarily improve udder health, and that isolated actions against the disease would not be able to replace a robust mastitis control or udder health promotion program [20,62,63]. Only the multifactorial understanding of the disease in each farm offers the theoretical-practical elements for designing specific programs for each alteration of the udder health [2]. Inconsistencies and the fragmented view of the interrelationship between risk factors may reflect a lack of knowledge of consequences for preventive medicine [22]. Therefore, the specific characteristics of each production system must be studied to plan and execute effective measures to improve udder health.

### Genetics, resistance, and immunity category

This category assumes that udder health is conditioned by genes and environment, which ultimately, defines udder phenotype, that is, the group of traits that promote greater susceptibility to the disease or the resistance a cow has against intramammary infection by bacteria.

Heritability studies are carried out to define whether the traits related to the health of the udder are more conditioned by genetics, by the environment, or by the interaction between these two factors. This type of study aims at designing and implementing strategies for animal selection. One study proposed an alternative approach to describe udder health using analysis traits (number of treatments for mastitis, number of disease periods, and number of days with the disease) [64]. Another study focused on features related to the conformation of the udder and udder health indicators (anterior udder insertion, udder depth, udder cleft, udder balance, udder conformation, front teat placement, milk yield, fat, % fat, protein, % protein, clinical mastitis, and SCC) [65]. A similar study analyzed the SCC traits for the first three lactations and udder type traits (front udder insertion, rear udder height, rear udder width, udder cleft, udder depth, front teat location, back teat location, and front teat length) [66]. Together with udder traits, interest has been shown in the analysis of features related to the hoof and legs of the cows in production (width of the rear udder, location of back and front teats, depth of the udder, texture of the udder, central ligament, fixation of the anterior part of the udder, teat length, height of the posterior part of the udder, angle of the hoof, depth of the heel, parallel hind legs, and sickle hind legs) [67]. Regarding the environment surrounding the cows, it has been found that features such as SCC, fat production (mainly C18: 1 cis-9), may be conditioned by heat stress [68]. In that study, a greater sensitivity to heat conditions was recorded in temperate regions, which suggests that genetic selection needs to be considered together with both phenotype of the animals and meteorological records in the area [68].

In a study in Iran [69], the traits of interest were days to first heat, days to the first service, the interval from calving to conception, the interval between calvings, and the number of inseminations per cow (reproduction traits); mastitis (udder health trait), mean lactation SCC, mean logarithmic transformed monthly SCC, mean of the transformed logarithmic monthly SCC, the standard deviation of the monthly SCC (binary traits); and fat: protein ratio (energy status trait). In that study, udder health traits had low heritability; however, there was genetic variability that implied the possibility of selecting superior animals because of this characteristic [69]. According to the results of another study [70], the existence of moderate positive genetic associations between milk fat, protein ratio, the interval between calvings, and udder health traits is plausible. This may indicate that cows with a relatively higher milk fat: protein ratio tend to have longer intervals to first insemination post-calving and to have increased susceptibility to mastitis due to negative energy balance (NEB) and subsequent mobilization of body reserves during early lactation [70].

Three milk flow traits - average milk flow rate, maximum milk flow rate, and milking time - have been correlated with udder health traits. SCC was recorded monthly, and mastitis was recorded daily to establish the relationship between these traits. The results led to conclude that, to achieve a more uniform milking duration between cows and, simultaneously, to avoid deterioration of udder health it is crucial to exclude genetic selection from the selection program cows with lesser or greater milk flow rates compared to the average of the group [3].

Candidate genes for determining susceptibility to mastitis have been studied. Neuropeptide FF receptor 2 (NPFFR2), member 4 genes belonging to the SLC4 family (SLC4A4), deoxycytidine kinase (DCK), leukemia inhibitory factor receptor (LIFR), and endothelin 3 (EDN3) were the proposed candidate genes proposed by a study in Denmark [71]. The gen NPFFR2 encodes for the G protein-coupled neuropeptide receptor family activated by the NPAF (neuropeptides A-18-amide) and NPFF (F-8-amide) with anti-inflammatory activity. The single nucleotide polymorphisms (SNPs) that surround the SLC4A4 gene have a high correlation with clinical mastitis. The DCK gene is associated with somatic cell scores, which is an indicator of mastitis. The LIFR gene is a suppressor of breast cancer metastasis of the Hippo-YAP pathway and a prognostic marker of clinical mastitis. Finally, the EDN3 gene influences the activation of neutrophils during early immunity response against mastitis pathogens. In Ireland, an association was found between SCC and the SNPs of the candidate genes of the somatotropic axis - Insulin-like growth factor I, Growth hormone 1, and the growth hormone transmembrane receptor - which emphasized the diverse pleiotropic effects of genetic variants in this pathway [72]. In Germany, the paternally inherited haplotype of a genomic region in *Bos taurus* autosome 18 affected SCC during the early postpartum period. This could also contribute to other aspects of health and production traits, due to indirect effects on feed intake and metabolism of animals [73].

Inbreeding depression has been estimated for some functional traits in dairy cattle. Inbreed animals tend to arrive older at first calving, have wider calving intervals, fewer days on production, and increased risk of giving birth to stillborn calves than non-inbreed pairs [74]. Inbreeding significantly affects the resistance of dairy cows to mastitis, as indicated by increased SCC in the first lactation and greater incidence of mastitis in the first three lactations [74]. This relates to the effect inbreeding has on economic return, as mastitis is the costliest infectious disease for the dairy industry. These findings also demonstrate the importance inbreeding has on milk production systems and udder health.

Regarding immunology of the udder, it has been found that blood serum proteins can be inflammation indicators, although they cannot be used to differentiate udder health states [75]. Stress conditions, such as those associated with NEB during early lactation, can alter the metabolic status and challenge the immune system, causing increased production of immunoglobulins and albumins in postpartum cows [76]. Alterations in the energy balance of cows have also been associated with an increased risk of developing IMI [76]. Ketone bodies in the blood are direct indicators of energy balance. Greater concentrations of B-Hydroxybutyrate in the blood are common on early postpartum high milk yield cows, especially when carbohydrates are deployed. This ketone body is used during the diagnosis of ketosis because it is stable in serum, plasma, and milk. The results of the study conducted by Cecchinato *et al*. [76] showed additive genetic variation for blood indicators of animal welfare and udder health traits, which could be exploited for the genetic improvement of the robustness of cows.

The immune response of the udder could be influenced both by the immune status of the cow and by external factors such as kind of pathogens and dairy management. Some explorations of this concept include the prevention of the reduction of immune defenses, particularly in heifers, which could be achieved through methods to increase the activity of the immune system (e.g., to increase the activity of the polymorphonuclear cells) [77]. Another article focused on the comparison of dairy breeds and their relationship with udder health [78]. The study compared the Holstein Friesians, Norwegian Reds, and their crosses concerning the health of the udder and its immune response. The results highlight the superiority of the Norwegian Reds breed over the other breeds evaluated for udder health. Furthermore, it was identified that crossing with Norwegian Reds improves udder health compared to pure breed Holstein Friesians [78]. In the same area, immunomodulators have been studied. A study conducted by Gulbe *et al*. [79] suggest that composition LGG (intramammary infusions of lysozyme, lactic acid, and glycopeptides) isolated from *Lactobacillus* spp., modulate T line of lymphocytes and the expression of lymphocyte activation markers, part of the adaptive immune system. This immunomodulator reduced the prevalence of pathogenic bacteria (*S. aureus* and the Enterobacteriaceae family) in milk by 75%, suggesting that the LLG composition could be used as an alternative treatment for subclinical udder infection in cows [79].

### Animal welfare, nutrition, and organic production categories

These categories understand udder health from a conceptual proposal based on health promotion. Animal welfare studies reaffirm the multifactorial nature of mastitis, where in addition to cow characteristics and management factors, the human-animal relationship is relevant to udder health. It has been found that workers’ positive behavior during milking was associated with a lesser SCC and a lesser prevalence of quarters undergoing mastitis. The occurrence of new infections appears to be more influenced by management factors than by the human-animal relationship. However, the human-animal relationship, especially human behavior during milking, should receive more attention as a possible intervention point on extension programs to prevent mastitis [5]. In the same line of theoretical construction, the behavior of dairy producers with cows can not only cause stress in the animals but also reduce it. A study in Germany evaluated the Tellington TTouch^®^ method (Linda Tellington-Jones, Canada), a gentle contact method based on light movements of the fingers and hands when circularly moving the skin to assess if this handling of heifers just before calving caused a reduction of avoidance distances, less agitation during milking, and lesser SCC in early lactation [80]. However, the researchers could not detect the positive effects of this method on avoidance distances and shaking behavior during milking, nor for SCC within the first 100 DIM compared to control heifers [80]. Fulwider *et al*. [81] found a correlation between closer voluntary approaches of cows to humans in the milking parlor and a lesser SCC.

The conceptual category nutrition proposes the promotion of udder health from the inclusion of vitamins, minerals, herbs, among other nutrients in the diet of dairy cows. The concentration of selenium in the blood plays an essential role in maintaining udder health. Results indicate that 180 μg of Selenium/liter of whole blood (Se/L) appear to be critical for all infections and the prevention of *S. aureus*, CNS, *Actinomyces pyogenes*, or *Corynebacterium* spp. Vitamin E, Vitamin A, and carotene in concentrations >4, >0.4, and >3 mg/L, respectively, appear to be sufficient to optimize udder health, improve the immune response of cows, and decrease clinical signs of infection [4]. In agreement, in Slovakia, Vasiľ *et al*. [82] found that the repeated parenteral administration of a product containing Vitamin E and Se to pregnant dairy cows showed a positive effect on the immune response, increasing the concentrations of alpha-tocopherol and Se in plasma and colostrum, as well as a reduction of clinical forms of mastitis post-calving. Selenium-deficient cows had greater SCC, exhibited inflammatory processes in the udder (stronger immune infiltration of parenchymal cells), inflammation in the parenchyma of the mammary gland (acinar atrophy, connective tissue proliferation, and granuloma formation) [83]. When included selenium in the diet, the prevalence of intramammary infection and SCC decreases [84-86]. Other minerals have also been used as supplements in the diet of dairy cows to promote udder health. Among the minerals, the action of Clinoptilolite zeolite (CPL) and Zinc (Zn) has been evaluated. One study [87] identified that cows that did not receive supplementation with CPL had 21 times more probability of intramammary infection than cows supplemented with CPL. This result can be attributed to CPL’s antibacterial, detoxifying, antioxidant, and immunostimulating effects on cow metabolism, evidenced by a decreased incidence of intramammary infections during the dry period, parturition, and early lactation for the supplemented cows [87]. According to a Serbian study [88], the optimal cutoff for Zn that separates healthy cows from cows with subclinical mastitis was 9.64 mmol/L. The role of Zn concentrations on udder health is crucial since cows with low concentrations of Zn had a greater SCC, a thinner stratum corneum in the skin of the teats, and an intense infiltration of leukocytes in the parenchyma of the udder [88].

The relationship between indigenous traditional knowledge and udder health has also been evaluated. In India, a raw polyherbal preparation supplement based on *Withania somnifera, Ocimum sanctum, Tinospora cordifolia, Emblica officinalis, Nigella sativa, Tribulus terrestris*, and *Asparagus racemosus* was validated on peripartum immunity and udder health in Karan-Fries crossbred cows [89]. The results suggest that this preparation can be used as an additive to improve immunity and, therefore, decrease the prevalence of subclinical mastitis and the incidence of clinical mastitis [89]. Similar results were obtained with a mixture of herbs rich in phytobiotics. The supplementation of these herbs effectively reduced SCC and improved performance in mid-lactation cows with a moderate or increased SCC, suggesting an improvement in their udder health [90]. Concentrated pomegranate extract - *Punica granatum* (CPE) has been evaluated with similar results to the other studies previously described in this category. Holstein cows fed with 40 g CPE addition per kg total mixed ration dry matter (DM) were more resistant to clinical mastitis since SCC levels and the proportion of cows with increased SCC (>200,000 SCC/mL) showed a reduction [91]. In addition, the 40 g CPE/kg DM supplement increased milk yield, milk energy, and antioxidant levels in milk. Such effect was more apparent in cows undergoing chronic mastitis and in early lactation cows [91].

Organic farming has been proposed in this review as a conceptual category of the udder health concept focused on cleaner production and public health and as an alternative to reduce the use of antibiotics in milk production. It has also been suggested that prevention practices associated with organic milk production systems and the extension programs commonly developed on these farms can provide a robust protective factor for udder health [6,92]. Such programs make it necessary to identify specific problems and strategies for problem-solving under specific management at the farm level [6,92]. Some studies have shown that good management of udder health translates into no significant differences between organic and conventional production farms for some products and health variables [93,94]. The use of alternative veterinary treatments against mastitis, the cull rate due to udder health disorders [93], and the calving interval [94] were greater for organic farms than traditional ones. In Spain, greater SCC has been reported in organic farms compared to conventional farms; this is explained by an increased prevalence of IMI caused by the decreased use of antibiotics for prophylaxis and treatment of udder infections in organic farms [95]. In Germany, udder health was the main problem on organic dairy farms, despite all efforts made by the organic production sector to prevent disease implementing effective regulations.

Furthermore, contrary to consumer opinion, udder health in organic dairy farms is comparable to that of conventional farms, with a trend towards poorer udder health in some respects, despite animal health is a fundamental objective in organic production [96]. Primarily, the rate of new intramammary infections during the drying period, the incidence rate of clinical mastitis, and the rate of mastitis in heifers represent critical udder health problems in organic dairy farms [96]. Therefore, improvements in udder health are necessary.

A study by Busato *et al*. [97] found an increased prevalence of contagious udder pathogens and increased SCC in alpine organic dairies in Switzerland. The prevention and therapy methods carried out in Swiss organic systems were mainly based on conventional procedures, but the proportion of producers who used antibiotics for dry cow therapy was more significant than in regular organic dairy and the antibiotic resistance patterns were not very different from conventional systems [97]. The same types of mastitis-causing bacteria have been isolated from cows’ milk in organic and conventional systems, with CNS and *S. aureus* being the most prevalent bacteria in a Norwegian study [98]. This study also found a more significant percentage of clinical mastitis in conventional systems than in organic ones. However, similar bacteria were found in both systems but in different proportions (*S. aureus* 27.8 vs. 20.2% and *Escherichia coli* 23.6 vs. 19.1% for conventional and organic systems, respectively). Resistance to penicillin of CNS and *S. aureus* isolated from cows undergoing subclinical mastitis showed slight differences in traditional and organic systems (48.5 vs. 46.5% for CNS and 8.8 vs. 14.1% for *S. aureus* in traditional and organic systems, respectively) [98]. The comparison of antibiotic resistance between traditional and organic systems shows controversial results. The Norwegian study found no antibiotic resistance for *S. aureus* in isolates of clinical mastitis from organic and conventional systems. However, all isolates of CNS in organic systems and three out of five isolates of CNS in conventional systems were resistant to penicillin [98]. In contrast, it has also been reported that *S. aureus* isolates are more likely to resist penicillin in conventional systems [99]. However, the choice of antibiotics and the treatment cases are very different between countries, comparisons are difficult [98].

### Culture and politics categories

**Figure-3:**
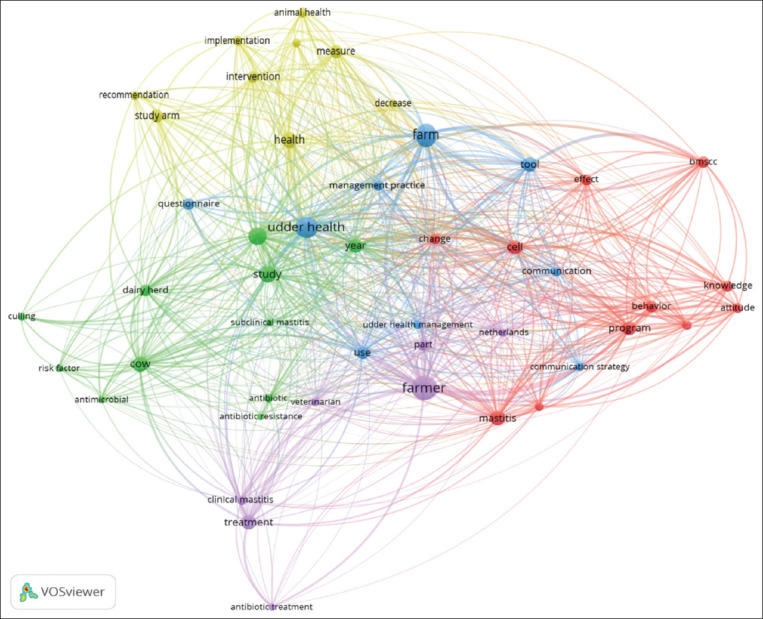
Networks of keywords in publications focused on culture and politics conceptual categories of the term udder health [11].

The scientific literature proposes culture as a conceptual category that contributes to constructing the concept of udder health. In this category, the milk producer is recognized as a study subject in the health-disease process and is proposed as one of the main components in udder health, understanding the individual’s self-awareness, behavior, and cosmovision (Figure-2). In this systematic review, the study subjects of the articles were the behaviors that lead to decision making related to the culling of dairy cows with chronic mastitis [100]; the beliefs, values, and motivations that define management practices in production systems [101]; the beliefs and attitudes toward the human-cow relationship and its impact on animal stress, animal welfare, promotion of udder health, and recovery from mastitis [102]; the mentality and behavior of the producers to adopt the strategies proposed in a public policy for udder health, to reduce of use of antibiotics and to decrease resistance to antibiotics, and to control of mastitis [7,12]; and the cultural characterization of the producers as a priority to establish communication strategies with veterinarians that promote udder health programs (Figure-3) [11].

The conceptual category politics has focused on analyzing some aspects of programs and public policies on udder health established in some countries, especially on understanding producers’ acknowledgment of such policies. In this category, the producer is recognized as a political actor in udder health promotion and in this sense, as one of the nuclei to understand the nodes in the socio-political networks established among actors of the dairy chain around this concept (Figure-3). Thus, this conceptual category includes the analysis of preventive health programs based on political elements, such as health planning about mastitis interventions and the rational use of antibiotics [103]; the implementation of herd health plans in organic dairy [104]; the evaluation of communication and motivation strategies in programs for udder health improvement, which showed to be more successful when the objectives of these strategies and the different motivations of the farmers are matched, based on the recognition of the cultural diversity of dairy producers [105]; the achievements in control of risk factors in the context of a policy of restricted use of antibiotics in dairy farming [106]; processes of sensitization, adoption of recommendations for decision-making, proactivity of dairy farmers to participate in udder health programs, compliance with rules, and the initiative of producers to adopt rational use of antibiotics [107]. As part of the political process, the collaboration between actors in the dairy industry has been registered [108]. Dairy companies and farmers have implemented tools, strategies, and interventions, demonstrating that microbiological indicators of commercial milk could be improved, as well as social influences, collaboration, communication networks, and health plans to promote udder health [108]. These same political processes have been studied, focusing on the social impact of veterinarians and their use of diagnostic tools that allow the establishment of effective diagnostic protocols. Thus, positive changes have been reported in the management of udder health, the treatment of mastitis, and the prevention of resistance to antibiotics [109]. Finally, additional aspects of the political category have been registered [8]. These aspects comprehend social norms, the establishment of trust and reciprocity norms between dairy farmers, and the acceptance of recommendations provided by dairy leaders and veterinaries [8]) (Figure-3).

In the graph, udder health and producer stand out as the most concurrent words, forming the nucleus of the central nodes that establish the possibility of a greater understanding of the meaning of udder health, from their cultural and political categories which, as human processes, are inseparable for udder health study.

### Proposed construct for the concept udder health

Based on the theoretical synthesis of each category and the uses of the term udder health, we propose the following construct based on the categories developed theoretically:

Udder health refers to the health-disease process in dairy production systems with implications for productivity, animal welfare, and public health. This dynamic and complex process is mediated by dairy producers and dairy industry human networks and requires to be understood from the interaction of many knowledge categories. Within the category traditional epidemiology based on risk factors and disease, the understanding of udder health is based on risk factors for the disease and from the knowledge of the disease itself. These risk factors are grouped in anatomic and physiological characteristics, factors related to milking and production, health-related practices, environmental risk factors, identification of pathogens, and antibiotic susceptibility. Together with the microbiology one, this category has allowed acceptable management practices for milk production and udder health to be established, which is mainly focused on preventive actions as best intervention practices against the disease. Complementing the construction of the two former categories, the categories genetics, resistance and immunity, nutrition, animal welfare, and organic production propose an approach oriented towards the promotion of health, the right to health of animals, and a cleaner production aiming at consumer care. Genetics, resistance, and immunity assume that udder health is determined by genes and environment, which determine udder phenotypes, thus promoting greater susceptibility or resistance to IMI caused by microorganisms. The category animal welfare contributes theoretically to the udder health concept from its understanding of the human-animal relationship and producers’ behavior towards the cows to reduce cow stress. The category nutrition conceptualizes the effect feed supplements have on udder health. Stimulation of immune response against mastitis, regulation of inflammation, beneficial antibacterial effects, detoxification, antioxidative effects, and how supplements can increase milk yield and energy contained in milk can be counted among these effects. The category organic production has focused on cleaner production and public health, suggesting alternative production practices accompanied by extension programs, alternative veterinary treatments to antibiotics, and the need for a particular diagnosis of each farm for decisions making on udder health programs.

These categories are based on a deterministic philosophical orientation that limits the analysis of phenomena to their implications, based on causality, not considering each category as a fundamental part of a system, in this case, udder health. This study allowed us to understand the networks and relationships there are between the proposed categories.

In the past decade, it has been suggested that udder health alterations are a problem mediated by human beings. Categories culture and politics are thus transversal to all other categories and, from a social perspective using quantitative and qualitative methodologies, emphasize the human processes that lead to decision-making. These two categories help us understand why the practices of control, prevention, and health promotion achieve success or failure. Within the category culture, it has been understood that more than a set of technical skills for milk production and udder health practices, it is important to consider beliefs, values, attitudes, knowledge, and motivations of dairy producers that at, the end, create behaviors that promote of decrease udder health. Finally, the category politics has contributed to the concept of udder health by presenting dairy producers and dairy industry actors as social-political subjects that, based on communication, collaboration networks, the establishment of trust and reciprocity norms, the appropriation and implementation of informal and formal rules, and health planning decisions, improve or worsen udder health and, therefore, public health.

## Conclusion

Udder health is a polysemic concept made up of various categories. These categories are described in this systematic review individually, although the interrelationships established among them are clear and inseparable to achieve udder health in dairy systems. The udder health concept can be understood in traditional epidemiology categories based on risk factors and disease; microbiology; genetics, resistance, and immunity; animal welfare; nutrition; organic production; culture; and politics. Nevertheless, in the past decade, culture and politics have been two of the most researched theoretical developments, transversal to all other categories. This new integrative approach to udder health has allowed advances and establishment of strategies and policies focused on productivity, animal health and welfare, and the resignification of dairy farmers’ role in animal and public health in the countries where they have been developed.

### Study limitations

The scope of the conceptual categories for the concept of udder health found in the scientific literature collected and reviewed in this study may be limited by the terms included in the search protocol. Mastitis, for example, was not included, since the term only refers to the disease without encompassing the complexity of the health concept. In general, systematic reviews are limited by the number of articles considered in each of them compared to narrative reviews, which can include a higher number of studies that do not depend on inclusion and exclusion criteria. Systematic revisions are observational and retrospective studies that, by nature, may entail implicit biases.

### Scope of future studies

Future research is expected to propose guidelines for the reformulation of public policy related to milk production and commercialization in the various municipalities in the department of Antioquia (Colombia), based on the results of this study in relation to the concept of udder health. Likewise, future research is expected to validate the influence of the categories and subcategories presented in field studies.

## Authors’ Contributions

RZS, JFGM, and LAR: Conception and design. RZS, JFGM, and LAR: Data collection, analysis, and interpretation. RZS, JFGM, and LAR: Article writing. RZS, JFGM, and LAR: Final approval of the version to be published. All authors read and approved the final manuscript.
